# Diagnosis of Scrub Typhus

**DOI:** 10.4269/ajtmh.2010.09-0233

**Published:** 2010-03

**Authors:** Gavin C. K. W. Koh, Richard J. Maude, Daniel H. Paris, Paul N. Newton, Stuart D. Blacksell

**Affiliations:** Faculty of Tropical Medicine, Mahidol University, Bangkok, Thailand; Department of Medicine, University of Cambridge, Cambridge, United Kingdom; Center for Experimental and Molecular Medicine, Academic Medical Center, University of Amsterdam, Amsterdam, The Netherlands; Centre for Clinical Vaccinology and Tropical Medicine, Nuffield Department of Clinical Medicine, University of Oxford, Oxford, United Kingdom; Wellcome Trust–Mahosot Hospital–Oxford Tropical Medicine Research Collaboration, Mahosot Hospital, Vientiane, Lao People's Democratic Republic

## Abstract

Scrub typhus is transmitted by trombiculid mites and is endemic to East and Southeast Asia and Northern Australia. The clinical syndrome classically consists of a fever, rash, and eschar, but scrub typhus also commonly presents as an undifferentiated fever that requires laboratory confirmation of the diagnosis, usually by indirect fluorescent antibody (IFA) assay. We discuss the limitations of IFA, debate the value of other methods based on antigen detection and nucleic acid amplification, and outline recommendations for future study.

Scrub typhus (*Orientia tsutsugamushi* infection) is transmitted by the bite of larval trombiculid mites and is endemic to the land mass within the triangle bounded by Japan to the north, Northern Australia to the south, and the Arabian Peninsula to the west. Mortality in the pre-antibiotic era was variable and in some series, approached 60%,^1^ but specific and effective antimicrobial chemotherapy is now available.

Scrub typhus often presents as fever with little to distinguish it clinically from co-endemic diseases such as typhoid, leptospirosis, and dengue. The presence of an eschar supports the diagnosis but is variably present.^2^ Diagnosis, therefore, depends on clinical suspicion, prompting the clinician to request an appropriate laboratory investigation, and failure to diagnose the disease will likely result in treatment with ineffective β-lactam–based regimens.

The mainstay in scrub-typhus diagnostics remains serology. The oldest test in current use is the Weil–Felix OX-K agglutination reaction, which is inexpensive, easy to perform, and results are available overnight; however, it lacks specificity and sensitivity^3^ (Table 1). The indirect fluorescent antibody (IFA) test is more sensitive, and results are available in a couple of hours; however, the test is more expensive and requires considerable training (Table 1). IFA uses fluorescent anti-human antibody to detect specific antibody from patient serum bound to a smear of scrub-typhus antigen and is currently the reference standard.^4^ Indirect immunoperoxidase (IIP) eliminates the expense of a fluorescent microscope by substituting peroxidase for fluorescein (Table 1).^3^

All currently available serological tests for scrub typhus have limitations in which clinicians need to be aware, despite their widespread use. Although agreement exists that a ≥ 4-fold increase in antibody titer between two consecutive samples is diagnostic,^4^ such a diagnosis is retrospective and cannot guide initial treatment.

Diagnosis based on a single acute-serum sample requires using a cut-off antibody titer. Cut offs ranging from 1:10 to 1:400 are quoted, often with little corroborating evidence^4^ and without establishing titers in the healthy local population (necessary to distinguish background immunity from acute infection); that cut off is then used for all patients, irrespective of whether or not they come from a scrub-typhus–endemic environment. Although IFA may be modified to report separate IgG and IgM titers, there is no consensus on when this is useful or how to interpret the results. Currently available rapid bedside tests are based on serological methods and share the same inherent problems as IFA.

Most frequently, IFA uses antigen from just three serotypes: Karp, Kato, and Gilliam. Yet, enormous antigenic variation has been found everywhere where it has been sought.^5^ Eight different serotypes were found in mites from a single field in Malaysia.^6^ In South Korea, > 75% of isolates are of the Boryong serotype.^7^ On the Japanese island of Kyushu, > 90% of the disease reported is of the Kawasaki or Kuroki serotypes.^8^ The Infectious Disease Surveillance Center in Japan, therefore, recommends a two-pronged approach to diagnosis. First, local strains are included in the IFA for each prefecture; second, PCR of the blood clot is performed on all specimens,^8^ although buffy coat might be preferable. This recommendation is not widely implemented outside of Japan.^4^

Isolating *O. tsutsugamushi* requires biosafety level-3 facilities and culture on cell monolayers; median time to positivity is 27 days.^9^ Mouse inoculation is even more laborious and intensive on resources.^10^ Current methods of isolation are, therefore, not appropriate for the routine diagnosis of scrub typhus. There is an urgent need for alternative diagnostic methods; however, evaluation is hampered, because the current gold standard (IFA) is imperfect. In a Korean polymerase chain reaction (PCR) study of eschars, *O. tsutsugamushi* DNA was detected in six of seven patients who tested negative for scrub typhus by IFA but had eschars typical of scrub typhus.^11^ In a study from Thailand, 3 of 20 (15%) patients with fever had positive *O. tsutsugamushi* PCR, despite negative serology.^12^ One recent study attempted to surmount this problem by evaluating the proposed test against a panel of serological and PCR-based methods,^13^ but PCR is itself beset with problems. The high resource costs and training required make it impractical for many areas where scrub typhus is endemic (Table 1). That aside, the most appropriate specimen to use remains unclear. PCR of eschar material is more sensitive than blood and remains positive even after the initiation of treatment.^11^ Unfortunately, in a setting where eschars are present in 7% of patients,^2^ for example, eschar-based tests can have a maximum sensitivity of only 7%. Using buffy coat could improve sensitivity compared with whole blood,^13^ but blood-based assays are positive only during the time window of rickettsemia. It the optimal PCR target remains unclear; nested PCR targeting the 56-kDa antigen has been shown to be highly specific,^2^,^14^ but sequence variability may affect primer annealing and therefore, test sensitivity.^15^ A whole-blood–based assay targeting the 16S rRNA gene showed a sensitivity of only 37.5–52.3% (95% confidence interval) in real-world conditions^15^,^16^ (probably because median copy number was only 13 copies/mL of blood^16^), but it performed better than the 56-kDa gene target in the same study (sensitivity = 22.5–36.1%); however, this may have been caused by differences in the assay rather than differences in the target genes.^15^ The 47-kDa outer-membrane protein is highly specific for *O. tsutsugamushi*,^17^ and species-specific primers also exist for the highly conserved molecular chaperone gene, *groEL*.^13^,^18^ But, it remains to be seen if either target will allay concerns about detecting infection caused by previously undescribed serotypes.

Loop isothermal amplification (LAMP) is a technique for amplifying DNA that makes use of three specially designed primer pairs and the *Bst* DNA polymerase. There is no complicated DNA extraction procedure, and unlike PCR, the entire reaction takes place at the same temperature. This means that only a water bath or heating block is required, whereas PCR requires a thermocycler. The reaction is read visually (a positive reaction produces a white pellet) and does not require special equipment (Table 1).^13^ A small proof-of-principle study (nine patients) showed that LAMP could detect DNA levels as low as 14 copies/µL compared with 3 copies/µL for real-time PCR).^13^ However, the technique has yet to be validated in a prospective clinical trial.

Clinicians will remain dependent on serological methods until these issues are resolved, but work can be done to optimize their performance. Cut offs must be validated locally, and previously undiscovered serotypes must be assiduously searched for by examining rodents and chiggers, not merely patient isolates. We propose that new diagnostic assays not be validated against IFA alone but instead, be compared against a panel of both serological and antigen-detection assays (e.g., IFA with 47-kDA and/or 56-kDa PCR).

## Figures and Tables

**Table 1:** Comparison of the accuracy and performance characteristics of assays for acute diagnosis of scrub-typhus infection

Format	Assay	Acute sensitivity	Specificity	Cost/sample	Time	Ease	Setting	Comments
Isolation	*In vitro* isolation (cell culture)^9^	+	+++++	+++++	7–60 days	+	BSL3 reference laboratory	• Isolation of BSL3 agent • Requires infrastructure • Biocontainment issues • Retrospective diagnosis
Isolation	Mouse inoculation^10^	+	+++++	+++++	5–30 days	+	BSL3 reference laboratory	• Technically demanding • Isolation of BSL3 agent • Requires animal facilities • Biocontainment issues • Retrospective diagnosis
Serology	IFA^19^	++	+++	++++	2 hours	++	Reference laboratory/hospital	• Serology gold standard • Requires propagation and purification of BSL3 agents as antigen for assay • Requires fluorescence microscope • Standardization problems • Requires paired samples (retrospective diagnosis)
Serology	IIP^3^	++	+++	+++	2 hours	++++	Reference laboratory/Hospital	• Serology gold standard • Requires propagation and purification of BSL3 agents as antigen for assay • Requires light microscope only • Standardization problems • Requires paired samples (retrospective diagnosis)
Serology	Weil–Felix OX-K^20^	+	++	+	6–18 hours	++++	Primary hospital	• Poor sensitivity for acute disease • Requires paired samples (retrospective diagnosis)
Serology	Rapid point-of-care tests (e.g., Integrated Diagnostics Dip-S-Ticks)^20^	++	+++	+++	< 30 minutes	+++++	Primary hospital	• Does not require specialized equipment • Rapid and simple
Genetic	Real-time PCR (16S, 56 kDa, 47 kDa, *groEL*)^13^,^15^	+++	+++++	+++	3 hours	+++	Reference laboratory/hospital	• Expensive equipment • Requires infrastructure • Sensitivity dependent on sample type and timing • Possible contamination issues
Genetic	Loop amplification (*groEL*)^13^	+++	+++++	++	2 hours	++++	Primary hospital	• Simple • Inexpensive • Possible contamination issues

BSL3 = biosafety level 3; + = low/poor; +++++ = high/excellent on a five-point qualitative scale.
